# Uric Acid

**Published:** 1900-01-13

**Authors:** 


### The Hospital

A Journal of

The Medical Sciences and Hospital Administration.

Vol. XXVII.—No. 694.] EDITED BY SIR HENRY BURDETT, K.C.B., AND SOLOMON C. SMITH, M.D., M.R.C.P. [January 13, 1900.

### Uric Acid.

Hovering around the condition which we know as gout there are a number of others, which for general convenience we speak of as "goutiness," or describe still more vaguely in adjectival phrase as "gouty;" may be flying pains, or "muscular rheumatism," or asthma or eczema, or merely a sour stomach. For the past fifty years gout has been regarded as the result of what, in vulgar parlance, has been called an acid condition of the blood, or, more correctly, a lessened alkalinity of that fluid. As the result of the well-known observations of Sir A. Garrod on acute gout it has been generally agreed that whatever else may be true there at least is a retention of uric acid in the system, and that during a gouty attack the tissues are more highly charged than should be the case with this peccant material. Where this uric acid comes from is another question, and while some probably quite correctly regard it as the result of disordered metabolism, others take its presence in excess, and all the symptoms which that may cause, as merely proofs that the wrong sort of food has been taken. Hence much dogmatism as to diet. If a sprightly writer wished to make fun of the medical profession, perhaps nothing would give him wider opportunity for the exercise of his sarcasm than the extreme variety and the very opposite character of the *régime* which this, that, and the other medical authority has from time to time laid down for all varieties of mankind in regard to the dietetic management of the gouty state. Some see salvation in meat, others in milk, some advise fruit, others taboo all things containing sugar or tainted with acidity, while some wise men, eschewing all questions of hypothetical uric acid, and doubting greatly any direct connection between acidity of food or even of stomach and that assumed acidity of blood which is said to be at the bottom of the mischief, quiety investigate the habits of their patients, and prescribe what experience has shown to agree best with each of them. It has indeed to be admitted that we know very little about the *rôle* played by uric acid in the production of gout. The bubble of our conceit has been pricked by many recent observations, and the whole question is again in the air. We are now told by Dr. Chalmers Watson,[1] as the result of a careful series of investigations which he has recently made, that (1) the alkalinity of the blood is not diminished during an attack of gout, (2) the excretion of uric acid is not diminished during the attack, but the reverse, and (3) the amount of uric acid in the blood is not greater during the attack than in the intervening interval. After showing this he justly adds that, "if these points be accepted, we must start *de novo* in search of the cause of the acute paroxysm."

Nevertheless, uric acid is a sad reality to many, as every patient who excretes it in excess knows well enough, and whatever its relation to gout may be, one cannot doubt that the vital processes—the digestion, the assimilation, or the metabolism—of the man who deposits it in his tissues and excretes it in his urine, are abnormal. But even in regard to such objective symptoms as those related to the presence of uric acid, how diverse are the recommendations in regard to treatment! Why do we cut out red meat from our dietary? Why do we tell patients that they must not take sugar? Why do we allow fish, and draw the line at herrings and eels? Again, as Dr. Goodhart asks, in criticising one of the printed diet sheets which some physicians are so fond of giving to their patients, why should we object to fat? He says, "I see that fat stands next in this list of 'dreadfuls' . . . . but the wisdom of this exclusion seems to me to be cancelled by the allowance of butter in the 'may eat' and the exclusion of cream among the drinks." Yet these recommendations are often the outcome of clinical experience, and the treatment founded upon them is in a certain number of cases perfectly successful. We thus find ourselves face to face with the question whether to accept the conclusions of clinical observers with their so-called empirical recommendations, various and unreasoning as they may seem, or to give ourselves over to the scientific teachers who, founding their beliefs on chemical formulæ, would tie us down to uniformity of method?

The fact seems to be that uric acid is a sort of rubbish which is produced by the human organism even in perfect health, and in much larger degree when its inner workings are out of order. Its presence, however, gives but little guidance as to the exact process which is wrong, any more than an excess of

[1] Brit. Med. Jour., Jan. 6.

cinders in a stokehole tells whereabouts in the mill the extra strain is being put on.

Let us remember how different patients are the one from the other. Probably Dr. Goodhart is correct when he says that "the uric acid passer invariably presents to you a problem to be unravelled, and each case a problem more or less distinct and separate from every other. In one case the cause may be the visceral sluggishness that is the outcome of nervous exhaustion; in another the hereditary condition, whatever it be, that determines gout. . . . In another it may be a great or sudden shock, a great anxiety, an exhausting attack of illness; in another, some early defect of the kidney; in another, . . . . the first indication of growing old."

These things have to be inquired into. Clearly gout is no more uric acid than uric acid is a mere matter of diet. But both one and the other are indications that, from some cause, either hereditary or otherwise, the mill is not working properly. What is wrong must be sought for by investigating the machinery rather than by analysing the cinders.

